# “Low‐risk” myelodysplastic neoplasm (MDS): Time for a name change?

**DOI:** 10.1002/hem3.70066

**Published:** 2025-01-07

**Authors:** Shoshana Burke, Stephen P. Hibbs

**Affiliations:** ^1^ Barts Cancer Institute Queen Mary University of London London UK; ^2^ Wolfson Institute of Population Health Queen Mary University of London London UK

Disease names matter. Consider these historical changes in labelling: “gay‐related immune deficiency” (GRID) renamed as HIV/AIDS or “juvenile diabetes” renamed as type 1 diabetes. These name changes partially reflect better understanding of aetiology but such labels also shape the way diseases are perceived.

Here, we revisit the language used to describe myelodysplastic neoplasia (MDS). We review the rationale and consequences of renaming myelodysplastic *syndrome* as *neoplasia*. We then focus on the “low‐risk” designation of the majority of cases of MDS, arguing that this label is misleading and has real‐world consequences for patients, clinicians, and research funders.

## SYNDROME OR NEOPLASM?

The importance of nomenclature and perception of MDS was the subject of a recent World Health Organization (WHO) consortium.^1^ Historically, classification of MDS as neoplasms was controversial. However, MDS fulfils contemporary medical and biological criteria of cancer, and the recent WHO's proposal to rename MDS as “myelodysplastic neoplasms” aimed to “underscore their neoplastic nature and harmonise terminology.”^2^ A similar trend is seen for myeloproliferative neoplasms (MPN), where clinical discussions increasingly emphasise their status as blood cancers.

For MDS, the confusing compromise was to keep the acronym but change the name to myelodysplastic neoplasm.^3^ Importantly, the decision to change the name was well received by some patient support groups, who saw it as a clarification of the condition's status as cancer, rather than a change. Furthermore, designating MDS as a type of cancer provided access to cancer support groups and services within charitable blood cancer organisations.

However, renaming as myelodysplastic *neoplasm* risks causing unnecessarily distress for some patients.^4^ The psychological impact of the word cancer remains significant, freighted with ideas of toxic chemotherapy, hair loss, vomiting, and social isolation. It is biologically correct to classify MDS (and MPN) as neoplasms, but how this label plays out in the lives of individual patients is complex and highly variable.

## “LOW‐RISK” OF WHAT?

The nomenclature of “low‐risk” MDS is widely used in treatment guidelines,^1^, ^5^, ^6^, ^7^ scientific discussion, and clinical encounters. For pathologists concerned exclusively with the risk of progression to acute myeloid leukemia (AML), “low‐risk” is an apt designation. But progression to AML is not the only risk conferred by MDS, and the suffering of most MDS patients does not relate to progression. Despite a low risk of progression, “low‐risk” MDS has an average survival of just over 5 years.^8^ Furthermore, over 80% of patients suffer the life‐changing sequelae of anaemia, including fatigue, dizziness, and heart failure.^9^

Treatment options for “low‐risk” MDS are limited and add their own set of risks. Erythropoiesis stimulating agents (ESAs) are the only licensed first‐line treatment in the United Kingdom. ESA's invariably fail and patients eventually become dependent on blood transfusions, requiring an average of two units every 4 weeks.^10^ Even in healthcare systems where patients are not directly charged for their treatment, blood transfusions have financial consequences for patients, such as travel expenses to hospital and working day interruptions for blood tests and transfusion appointments. Transfusions confer risks of reactions, iron overload, and organ damage.^11^

## WHAT ARE THE CONSEQUENCES OF “LOW‐RISK” LABELLING?

The term “low‐risk” reflects the emphasis pathologists and clinicians place on disease progression to AML, rather than conveying the lived experience of most MDS patients.^12^ What are the consequences of the “low‐risk” label for the experience of patients and for the attitudes of clinicians and research funders?

At a recent meeting of the MDS UK Patient Support Group, three themes emerged. First, patients expressed a desperate need for new trials and treatments. Second, when experiencing symptoms and concern about their disease, patients found it unhelpful to be told that “no treatment is needed.” Third, patients can feel misunderstood or dismissed when told by a clinician that they have “one of the good cancers.” To a haemato‐oncologist, “low‐risk” MDS may appear a less devastating diagnosis than AML, but to a patient receiving the diagnosis, they may have no conception of AML to compare. A diagnosis of MDS does not feel “good” to the patient, and the “low‐risk” label can imply low burden.

Foregrounding the complex notion of progression “risk” can also create misunderstanding. When patients are told that no chemotherapy treatment is needed, some understand that nothing can be done for them, and progression to AML is inevitable. Twinges, headaches, and unexplained pains can make patients fear that their disease is progressing, but they may hesitate to contact their clinician, unsure if these symptoms warrant concern given their “low‐risk” designation.

Names are important, not just for the experience of individual patients but also in how they are viewed by healthcare professionals, researchers, and funders. The concept of “disease prestige” refers to the implicit hierarchy that exists in how different diseases are perceived by medical professionals and, often, society at large. Album and colleagues, who first introduced the idea of disease prestige, highlight several factors that elevate a disease's status.^13^ These include being acute and life‐threatening, involving high‐risk, high‐tech treatments, and affecting younger patients who accept medical explanations for their condition. In contrast, diseases that are chronic have limited treatment options, or affect older, more dependent individuals tend to be seen as lower status.

Does this notion play out within haematology? Think of the difference in your perception of a 72‐year‐old patient with “low‐risk” MDS and a 72‐year‐old diagnosed with myeloma. Both conditions are incurable and life‐limiting; however, while myeloma has a wide array of treatment options, “low‐risk” MDS is primarily managed with what is optimistically termed “best supportive care”: a euphemistic term that reflects a lack of effective treatment options.

The low disease prestige of “low‐risk” MDS may explain how why so little research has been conducted for these forms of the disease. Despite 68% of MDS patients being classified as “low‐risk,” only 6% of clinical trials are specifically for “low‐risk” disease.^14^ Disease prestige judgments affect priority setting within healthcare systems.^15^ The unintended depreciating effect of the “low‐risk” label creates barriers for clinicians and researchers in advocating effectively for their patients.

## LOSING THE “LOW‐RISK”

How might “low‐risk” MDS be renamed? It could be simplified to “MDS” in all cases—we accept heterogeneity in most other diseases without further defining them in discussions with patients. Alternatively, the name “Transfusion‐dependent MDS” for those on chronic transfusion programs would foreground the experience of anaemia symptoms and transfusion burdens. Removing the “low‐risk” label could helpfully shift perceptions and priorities. An individual's risk of disease progression to AML needs sensitive discussion with appropriate levels of reassurance but does not need to be in their diagnostic label.

Although MDS nomenclature may seem like a small aspect of broader clinical care, it affects the perception patients have of their condition and how they communicate this to others. Now that MDS is renamed myelodysplastic *neoplasm*, clinicians should consider the positive and negative implications for patients. Removing the “low‐risk” label can strengthen the case for addressing unmet needs through research and support clinicians to holistically discuss illness experience and treatment options.

## AUTHOR CONTRIBUTIONS

Shoshana Burke and Stephen P. Hibbs conceptualized the article and wrote the initial draft. Shoshana Burke and Stephen P. Hibbs critically reviewed the article and revised it. Shoshana Burke and Stephen P. Hibbs agreed to the final version.

## CONFLICT OF INTEREST STATEMENT

The authors declare no conflict of interest.

## FUNDING

Shoshana Burke is supported by a HARP doctoral research fellowship, funded by the Wellcome Trust (Grant number 223500/Z/21/Z). Stephen P. Hibbs is supported by a HARP doctoral research fellowship, funded by the Wellcome Trust (Grant number 223500/Z/21/Z). No funding was received for this publication.

## Data Availability

Data sharing not applicable to this article as no data sets were generated or analyzed during the current study.
